# New N-Alkylated Heterocyclic Compounds as Prospective NDM1 Inhibitors: Investigation of In Vitro and In Silico Properties

**DOI:** 10.3390/ph15070803

**Published:** 2022-06-28

**Authors:** Yassine Kaddouri, Btissam Bouchal, Farid Abrigach, Mohamed El Kodadi, Mohammed Bellaoui, Ahmed Elkamhawy, Rachid Touzani, Magda H. Abdellattif

**Affiliations:** 1 Laboratory of Inorganic Chemistry, Department of Chemistry, University of Helsinki, P.O. Box 55, FI-00014 Helsinki, Finland; 2 Genetics Unit, Medical Sciences Research Laboratory, Faculty of Medicine and Pharmacy of Oujda, University Mohammed Premier, Oujda 11022, Morocco; btissam.bouchal@gmail.com (B.B.); bmbellaoui@gmail.com (M.B.); 3 Laboratory of Applied Chemistry & Environment (LCAE), Faculty of Sciences, University Mohammed Premier, Oujda 11022, Morocco; f.abrigach@ump.ac.ma (F.A.); elkodadim@gmail.com (M.E.K.); r.touzani@ump.ac.ma (R.T.); 4 CRMEF Oriental, Center régional des Métiers de l’Education et de Formation, Oujda 11022, Morocco; 5 BK21 FOUR Team and Integrated Research Institute for Drug Development, College of Pharmacy, Dongguk University-Seoul, Goyang 10326, Korea; a_elkamhawy@mans.edu.eg; 6 Department of Pharmaceutical Organic Chemistry, Faculty of Pharmacy, Mansoura University, Mansoura 35516, Egypt; 7 Chemistry Department, Sciences College, Taif University, P.O. Box 11099, Taif 21944, Saudi Arabia

**Keywords:** synthesis, pyrazole, antibacterial, antifungal, ADME-Tox, molecular docking

## Abstract

A new family of pyrazole-based compounds (**1**–**15**) was synthesized and characterized using different physicochemical analyses, such as FTIR, UV-Visible, ^1^H, ^13^C NMR, and ESI/LC-MS. The compounds were evaluated for their in vitro antifungal and antibacterial activities against several fungal and bacterial strains. The results indicate that some compounds showed excellent antibacterial activity against *E. coli*, *S. aureus*, *C. freundii*, and *L. monocytogenes* strains. In contrast, none of the compounds had antifungal activity. Molecular electrostatic potential (MEP) map analyses and inductive and mesomeric effect studies were performed to study the relationship between the chemical structure of our compounds and the biological activity. In addition, molecular docking and virtual screening studies were carried out to rationalize the antibacterial findings to characterize the modes of binding of the most active compounds to the active pockets of NDM1 proteins.

## 1. Introduction

Resistance to antibiotics pushes researchers to discover new antibacterial candidates as prospective treatments for different infectious diseases with another class of antibiotics with specific mechanisms of action.

**Figure 1:**
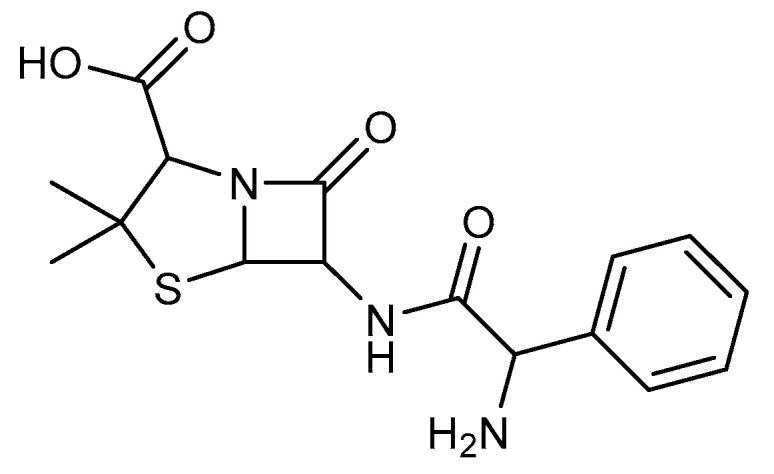
Chemical structure of Ampicillin.

Beta-lactam antibiotics [1,2], commonly known as penicillin-binding proteins (PBPs), act as mechanism-based inhibitors by targeting the cell wall-modifying DD-transpeptidases, susceptible to nucleophilic attack from long-lived acylated complexes. PBPs are responsible for the formation and integrity of the membrane surface’s rigid mesh-like peptidoglycan layer exterior. However, there is a mechanism of resistance to beta-lactam due to beta-lactamases. Where Ampicillin was selected as an example for this study, the selection was because it has an amine and carbonyl function similar to our first target compound (Figure 1) that binds to the penicillin-binding proteins. Commonly used broad-spectrum antibiotics are streptomycin [3,4,5,6,7,8,9] and cefotaxime [10,11], a third-generation cephalosporin [5,12,13,14] with less susceptibility to beta-lactamase [15,16,17] than ampicillin [5,18,19,20].

Infectious diseases are the main threat, especially in developing countries [21], and include listeriosis [4,22,23,24] caused by *L. monocytogenes*, septicemia, and meningitis caused by *E. coli* [8,25], bloodstream infections, and meninges caused by *C. freundii* [26,27,28,29]. These diseases commonly affect healthy, sensitive individuals, such as older people, pregnant women, and the immunosuppressed [11]. In addition, however, candidiasis and related fungal bloodstream infections are caused by *Saccharomyces cerevisiae* [30], *Candida albicans*, and *Candida glabrata* [31].

Pyrazole-based heterocyclic ligands have multiple biological applications. Many compounds prepared in our research group already have high efficiencies [32,33,34,35,36,37,38,39,40,41,42,43] as antibacterial or antifungal candidates [34,38,44,45,46] due to their nitrogen electron and proton acceptor abilities [32]. With limited facilities to investigate more experimental properties, molecular docking [43,47,48,49,50,51,52,53,54,55,56,57] becomes crucial for studying the binding modes and affinities between the prepared compounds and selected biological targets using the lock and key concept. In our study, various tripodal pyrazole ligands were prepared and characterized using FTIR, UV-visible, ^1^H, and ^13^C NMR, and then their toxicity predictions and the Lipinski rule of five agreement were determined. Finally, the molecular ligand-protein docking, through the New Delhi metallo β-lactamase hydrolysis of β-lactams antibiotics [17,54,58,59], was studied in two different active sites to investigate our studied compounds’ binding susceptibility to the hydrolase enzyme.

## 2. Results and Discussion

### 2.1. Antibacterial and Antifungal Activities

The antibacterial potential of the compounds against two Gram-negative bacterial strains (*Escherichia coli* and *Citrobacter freundii*) and two Gram-positive bacteria (*Staphylococcus aureus* and *Listeria monocytogenes*) was evaluated as described in the materials and methods section, and the results are displayed in Table 1. Only compounds **12** and **14** showed antibacterial activity when tested at 500 μM. Compound **12** was active against *E. coli*, *S. aureus,* and *C. freundii* but inactive against *L. monocytogenes,* whereas compound **14** was only active against *L. monocytogenes*.

The MICs of compounds **12** and **14** were then determined as described in the material and methods (Table 2). The MIC of compound **12** was 134.9 mg/L against *E. coli*, 168.7 mg/L against *S. aureus,* and 168.7 mg/L against *C. freundii.* For compound **14**, the MIC against *L. monocytogenes* was 134.6 mg/L. Interestingly, the determination of the MBC of these compounds showed that they are bactericidal, as demonstrated by the ratio of MBC/MIC ≤ 2 (Table 2).

Regarding the antifungal activity, all the compounds were tested for toxicity against *Saccharomyces cerevisiae* and two species of Candida, *Candida glabrata* and *Candida albicans,* as described in Section 3.3.3. All compounds showed no antifungal activity against all three strains used. Together with the antibacterial activity analysis, these results suggest that compounds **1** to **15** lack antifungal activity, and only compounds **12** and **14** act specifically as antibacterial agents.

### 2.2. MEP Analysis of the Compounds **12**, **14**, Ampicillin, and Cefotaxime

**Figure 2:**
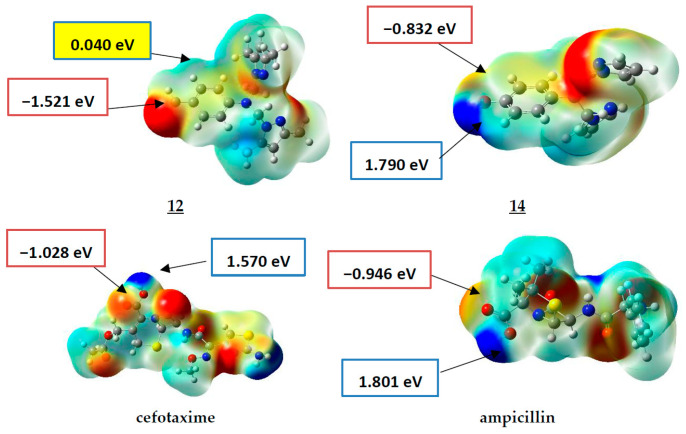
MEP surfaces of compounds **12**, **14**, ampicillin, and cefotaxime (−4.300 × 10^−3^ (Red) to 4.300 × 10^−3^ (Blue)).

Molecular electrostatic potential (MEP) maps of compounds **12**, **14**, Ampicillin, and cefotaxime have been generated (Figure 2) to determine and predict the reactive sites (nucleophilic or electrophilic) on the molecular system of the studied compounds.

As presented in Figure 2, the positive electrostatic potential areas (in blue) are concentrated over the hydroxyl group of the drugs cefotaxime and Ampicillin with 1.570 and 1.801 eV (at an iso value of 0.0004 electrons/Å^3^). For compound **14**, the highest value was about 1.790 eV (located on the hydroxyl group substituent on the phenyl ring), while a low value of 0.040 eV was estimated for compound **12**.

The negative electrostatic potential areas are located over the carbonyl group of the acidic function of the drugs cefotaxime and Ampicillin, with values of −1.028 and −0.946 eV, respectively, with a higher value for the compound **12,** with the value of −1.521 eV. In comparison, it was −0.832 eV for compound **14**.

To sum up, compound **12** only has a higher negative charge value over the carbonyl. In contrast, compound 14, similar to the antibiotics cefotaxime and Ampicillin, has lower negative–positive (δ^+^-δ^−^) charge values over the hydroxyl and the acidic function. These results, which agree with the experimental results, give us information about the possible sites for binding modes to the biological targets that need molecular docking investigations.

**Figure 3:**
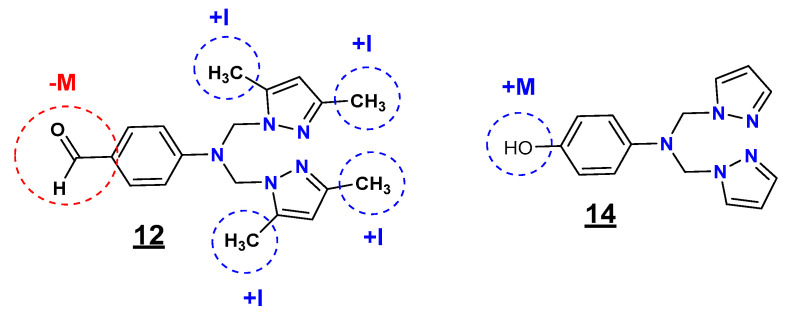
Inductive and mesomeric effect study of the compounds **12** and **14**.

In general, and in the light of these observations, we can postulate that the substitution by groups with different electronic effects (withdrawing-donating) is more beneficial in improving the antibacterial potency than the substitution by one group (Figure 3).

Regarding the R_1_ (substituent on the phenyl ring) and R_2_ (substituent at positions 3 and 5 of the pyrazole moiety) substituents, the inductive and mesomeric effect study revealed that the presence of the formyl (CHO) group (electron-withdrawing effect (-M)) on the phenyl ring and the methyl (CH_3_) groups (electron-donating effect (+I)) on the pyrazole moieties at positions 3 and 5 (**12**) is highly favorable for the inhibitory potency against *E. coli, S. aureus,* and *C. freundii* strains. In contrast, the presence of the hydroxyl (OH) group (electron-donating effect (+M)) on the phenyl ring with non-substituted pyrazole moieties (**14**) resulted in selective antibacterial activity against *L. monocytogenes*.

### 2.3. ADME and Toxicity Predictions

#### ADME Predictions

For the ADME predictions, the physicochemical properties (MW: molecular weight expressed in Daltons; logP: octanol/water partition coefficient characterizing Lipophilicity; HDO: number of hydrogen bond donors; HAC: number of hydrogen bond acceptors; NRO: number of rotatable bonds; TPSA: total polar surface area) were calculated and are presented in Table 3 for the compounds **12** and **14** and the drugs streptomycin, Ampicillin, and cefotaxime as references.

In Table 4, compounds **12** and **14** have no violations of Lipinski’s rule of five [58,59], with MW = 337.42 and 269.30 ˂ 500, logP value of 3.0933 and 1.227 ˂ 5, H donor of 0 and 1 ˂ 5, H acceptor of 3 and 3 ˂ 10, number of rotatable bonds of 6 and 5 ˂ 10 and TPSA value of 55.95 and 59.11 Å^2^ ˂ 140 Å^2^. This comparison highlights that the two compounds **12** and **14** have better oral bioavailability than Ampicillin and are better than streptomycin.

These results make compound **12** a better antibacterial candidate than cefotaxime with the same selective multitarget activity, but further toxicity predictions are required to validate these propositions.

### 2.4. Molecular Docking and Virtual Screening Studies

Ligand–protein docking simulations were carried out to determine the binding mode of the studied compounds with the catalytic sites of the selected receptors. Flexibility was allowed in all the rotatable bonds of the ligand; the protein was used as a rigid structure.

#### 2.4.1. Docking against the NDM-1 β-lactamase (NDM1) Protein

NDM-1 β-lactamase hydrolysis docking study of the compounds **12** and **14** compared to Ampicillin.

**Figure 4:**
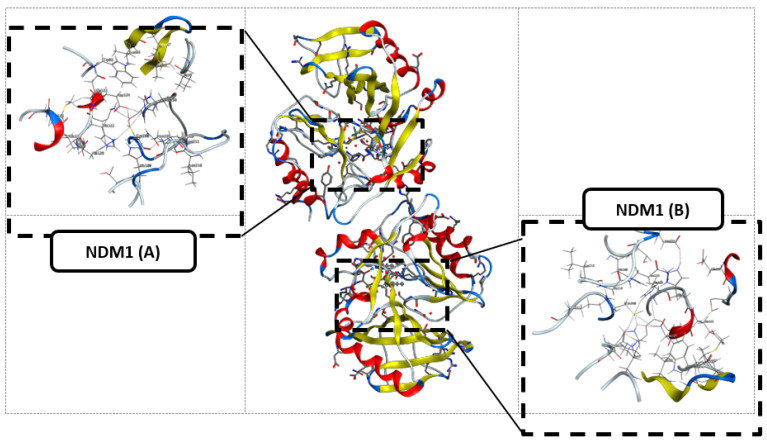
The three-dimensional structure of New Delhi metallo-β-lactamase (NDM1) and the two selected active sites.

The three-dimensional structure of New Delhi metallo-β-lactamase (NDM1) [17,58,59] is represented in Figure 4, where two sequences, A and B, were co-crystalized with Ampicillin; so in this part, we studied the binding affinity of the compounds **12** and **14** compared to Ampicillin.

The protein preparation was performed by removing all the water, zinc, and OH molecules.

As previously mentioned in the docking study of the transpeptidase inhibition study, the NDM1 hydrolysis followed the same parameters, and the binding affinity results of both active sites are collected in Table 4.

From Table 5, compound **12** had a better affinity than compound **14** in both selected active sites, NDM1 A and B, with a binding affinity of −6.0075 and −6.6776 Kcal/mol, respectively. In addition, compared to Ampicillin, with −6.9737 and −6.7344 Kcal/mol, compound **12** was more readily hydrolyzed than compound **14**.

**Figure 5:**
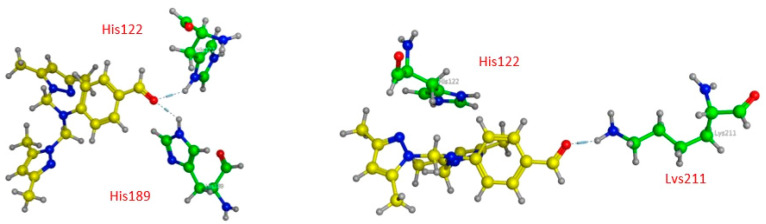
Three-dimensional presentations of the binding modes between the compound **12** and NDM1.

First, compound **12** has two H-acceptors, bond 3.08 and 2.90 Å, respectively, between the carbonyl and the nitrogen ND1 and NE2 of His122 and His 189, as shown in Table 5 and Figure 5.

**Figure 6:**
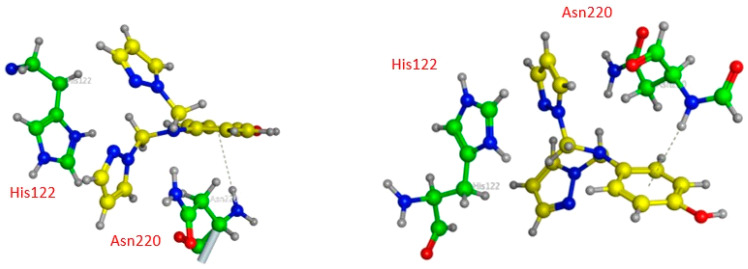
Three-dimensional presentations of the binding modes between the compound **14** and NDM1.

Contrary to compound **12**, compound **14**, as represented in Figure 6, has only weak van der Waals bonds with Asn 220 and His 122, with a distance range of 3.60–3.90 Å, making this compound more stable against NDM1 hydrolysis.

**Figure 7:**
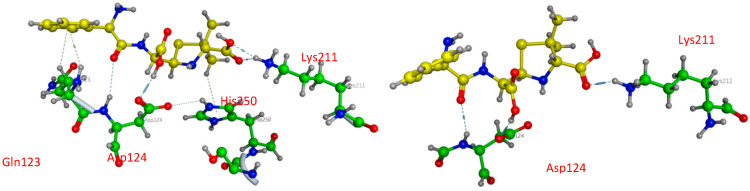
Three-dimensional presentations of the binding modes between Ampicillin and NDM1.

As penicillin β-lactam antibiotics reported in the literature, Ampicillin seems to interact with more residues at the site (A) than at the site (B) of NDM1 protein, as represented in Table 5 and Figure 7. In the active pocket A, the compound forms seven bonds: one H-donor and one H-acceptor (of 2.90 and 3.55 Å, respectively) with Asp124 residue, two H-acceptor bonds with Lys211 amino acid with distances equal to 2.98 and 3.37 Å, one H-pi interaction (4.07 Å) between the carbon (C16 12) atom and 5-ring of His250, and two pi-H interactions between the 6-ring and the nitrogen and carbon atoms of Gln123 (4.08 and 3.78 Å). In contrast, Ampicillin was found to bind into the active pocket B of NDM1 only with three interactions: two by H-donor and H-acceptor bonds with Asp124 (2.99 and 3.55 Å) and one by an H-acceptor bond with Lys211 with a distance of 2.88 Å involving its oxygen atom (O1 1).

From the docking study results, compound **12** is more sensitive to NDM1 hydrolysis, which inactivates its antibacterial activity against *Listeria monocytogenes* due to the common carbonyl function in all β-lactam antibiotics. Although otherwise, compound **14** has listericidal activity due to its weak binding with the selected NDM1, its specific mechanism needs more computational studies and biological assays for prediction.

#### 2.4.2. Blind Docking/Virtual Screening against the NDM-1 β-lactamase (NDM1) Protein

**Figure 8:**
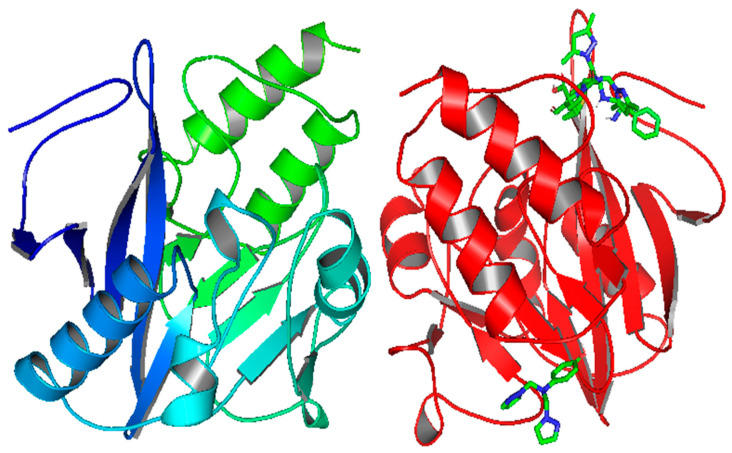
Docking poses of the ligands **12**, **14,** and **ampicillin** in the NDM1 protein (PDB: 5ZGE).

As presented in Figure 8, the B-chain has considered the active site with all the docking poses. At the same time, there is good alignment between the ligand **12 ** and ampicillin docking poses with smooth variation, while ligand **14** is so far in a different site.

Table 6 shows that the mode of binding interaction is the same as that of LYS216, which is bound with the nitrogen of pyrazole for the ligand **12** and the oxygen for Ampicillin with the same binding affinity of −7.1 Kcal/mol. On the other hand, ligand **14** has a lower −7.0 Kcal/mol value with amino acids such as ILE203 and LYS242.

## 3. Materials and Methods

### 3.1. Analytical Procedures

A Bruker DPX 800 MHz Spectrometer recorded the ^1^HNMR (500 MHz, DMSO-*d*6) and ^13^CNMR (125 MHz, chloroform-*d)* spectra. Chemical shift (**δ**) values were stated in parts per million (ppm) using internal standard tetramethylsilane, according to the D_2_O exchange. Chemical shift (d) values were stated in parts per million (ppm) using internal standard tetramethylsilane. The D_2_O exchange confirmed the exchangeable protons (OH and NH). The FTIR analyses were performed using an FTIR 8400S spectrophotometer recorded in KBr pellets.

Many different pyrazole derivatives were synthesized and indexed.

### 3.2. Chemistry

**Figure 9:**
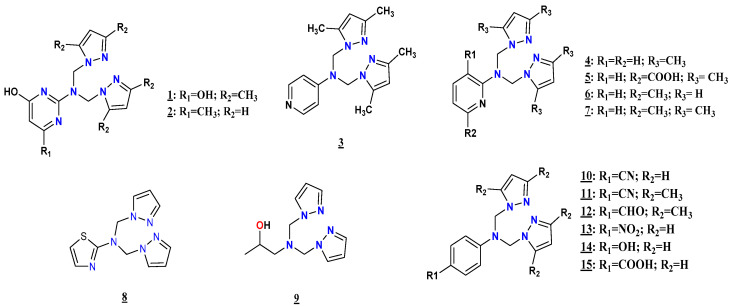
Structures of the compounds **1**–**15**.

The pyrazole derivatives (Figure 9) investigated in this work were prepared following the experimental procedure of the *N*-alkylation reaction described previously in the literature [43,46,60,61,62,63,64,65,66,67,68,69,70,71,72,73,74,75,76,77]. First, all the compounds were prepared by condensation of primary amines with (3,5-dimethyl-*1H*-pyrazole-1-yl)methanol or (*1H*-pyrazole-1-yl)methanol in acetonitrile as a polar aprotic solvent that promotes SN2 reaction; after that, the compounds were purified either by diethyl ether or a DCM:water (3:1) mixture to obtain the final products, with yields varying from 15.22 to 99.41%.

2-(Bis((3,5-dimethyl-*1H*-pyrazol-1-yl)methyl)amino)pyrimidine-4,6-diol (**1**)

2-Aminopyrimidine-4,6-diol (1 g, 7.86 mmol) and (3,5-dimethyl-*1H*-pyrazol-1-yl)methanol (1.98 g, 15.72 mmol) were mixed together in acetonitrile (20 mL) under reflux for 4 h, and the solvent was evaporated, then recrystallized in diethyl ether, and then filtrated to obtain the final product (1.28 g, 47.4%), mp > 250 °C (diethyl ether); FTIR (KBr, cm^−1^): 3348 (-OH); 3149 (C-H); 1684 (C=C); 1560 (C-C); 1455 (C-N); 1266 (C=N); 1067 (N-N); 779 (=C-H); ^1^H NMR (DMSO-d_6_, 500 MHz) δ ppm: 6.47 (s, 4H, H-CH_2_); 5.76 (s, 4H, H-OH and Hpyrz-4)); 5.25 (s, 1H, Hpyrm); 2.14 (s, 12H, Hpyrz), ^13^C NMR (DMSO-d_6_, 125 MHz) δ ppm: 105.46 (Cpyrz); 70.38 (CH_2_); 10.75 (Cpyrz-5); 10.19 (Cpyrz-3). The elemental Analysis was calculated for C_16_H_21_N_7_O_2_ (M.wt 384.13); C-55.96; H-6.16; and N-28.55 were the % calculated. C-55.81; H-6.06; and N-28.55 were the % found.

2-(Bis((*1H*-pyrazol-1-yl)methyl)amino)-6-methylpyrimidin-4-ol (**2**)

2-Amino-6-methylpyrimidine-4-ol (1 g, 7.99 mmol) and (*1H*-pyrazol-1-yl)methanol (1.57 g, 15.98 mmol) were mixed together in acetonitrile (20 mL) under reflux for 4 h, and the solvent was evaporated, then recrystallized in diethyl ether, and then filtrated to obtain the final product (3.8 g, 91.22%): mp ˃ 250 °C (diethyl ether); FTIR (KBr, cm^−1^): 3328 et 3069 (O-H Free and linked); 2925 (C-H); 1656 (C=C); 1493 (C-C); 1383 (C-N); 1172 (C=N); 1049 (N-N); 763 (=C-H); ^1^H NMR (DMSO-d_6_, 400 MHz) δ ppm: 10.53 (s, 1H, H-OH); 7.61 (d, 2H, Hpyrz-5, *J_H-H_* = 4–6 Hz); 7.30 (d, 2H, Hpyrz-3, *J_H-H_* = 4–6 Hz); 6.10 (m, Hpyrm-4); 4.82 (s, H-CH_2_ and Hpyrm); 1.91 (s, CH_3_); ^13^C NMR (DMSO-d_6_, 100 MHz) δ ppm: 137.65 (Cpyrz-3); 128.99 (Cpyrz-5); 104.67 (Cpyrz-4); 104.59 (Cpyrm); 73.69 (CH_2_); 21.59 (CH_3_).).The elemental Analysis was calculated for C_9_H_9_N_3_O_4_S (M.wt 255); C-54.73; H-5.30; and N-34.37 were the % calculated. C-54.65; H-5.21; N-34.29 were the % found.

*N,N*-bis((3,5-dimethyl-*1H*-pyrazol-1-yl)methyl)pyridin-4-amine (**3**) [61]

4-Aminopyridine (0.5 g, 5.31 mmol) and (3,5-dimethyl-*1H*-pyrazol-1-yl)methanol (1.34 g, 10.62 mmol) were mixed together in acetonitrile (20 mL) under reflux for 4 h, and the solvent was evaporated, then recrystallized in diethyl ether, and then filtrated to obtain the final product (0.48 g, 29.04%): mp 100–102 °C (diethyl ether); FTIR (KBr, cm^−1^): 2359 (C-H); 1648 (C=C); 1559 (C-C); 1454 (C-N); 1310 (C=N); 1071 (N-N); 807 (=C-H); ^1^H NMR (CDCl_3_, 500 MHz) δ ppm: 7.66 (d, 1H, Hpyrn-3, *J_H-H_* = 5–6 Hz); 7.24 (d, 1H, Hpyrn-2, *J_H-H_* = 5–6 Hz); 6.24 (s, 1H, Hpyrz-4); 5.66 (s, 2H, H-CH_2_); 2.34 (s, 3H, CH_3_^−^5); 2.09 (s, 3H, CH_3_-3); ^13^C NMR (CDCl_3_, 125 MHz) δ ppm: 167.73 (Cpyrn-1); 156.11 (Cpyrn-2); 140.41 (Cpyrz-3); 138.13 (Cpyrz-5); 113.78 (Cpyrn-3); 105.50 (Cpyrz-4); 57.61 (2H, CH_2_); 14.02 (Cpyrz-3); 10.96 (Cpyrz-5).

*N,N*-bis((3,5-dimethyl-*1H*-pyrazol-1-yl)methyl)pyridin-2-amine (**4**) [78,79]

2-Aminopyridine (0.5 g, 5.31 mmol) and (3,5-dimethyl-*1H*-pyrazol-1-yl)methanol (1.34 g, 10.62 mmol) were mixed together in acetonitrile (20 mL) at room temperature for 4 days, and the solvent was evaporated, then recrystallized in diethyl ether, and then filtered to obtain the final product (1.51 g, 92.13%): mp 88–90 °C (diethyl ether); FTIR (KBr, cm^−1^): 1609 (C=C); 1530 (C-C); 1423 (C-N); 1291 (C=N); 1067 (N-N); 772 (=C-H); ^1^H NMR (CDCl_3_, 500 MHz) δ ppm: 8.01 (d, 1H, Hpyrn-3, *J_H-H_* = 5–6 Hz); 7.31 (d, 1H, Hpyrn-4, *J_H-H_* = 4–6 Hz); 6.54 (dd, 1H, Hpyrn-5, *J_H-H_* = 4–6 Hz and *J_H-H_* = 5–6 Hz); 6.45 (d, 1H, Hpyrn-2, *J_H-H_* = 4–6 Hz); 6.38 (d, Hpyrn-6, *J_H-H_* = 5–6 Hz); 5.67 (s, 1H, Hpyrz-4); 5.50 (s, 2H, CH_2_); 2.35 (s, 3H, CH_3_-5); 2.27 (s, 3H, CH_3_-3); ^13^C NMR (CDCl_3_, 125 MHz) δ ppm: 156.65 (Cpyrn-1); 148.42 (Cpyrn-2); 147.41 (Cpyrz-3); 139.81 (Cpyrz-5); 137.49 (Cpyrn-4); 114.17 (Cpyrn-3); 109.03 (Cpyrn-2); 106.16 (Cpyrz-4); 54.33 (2H, CH_2_); 13.43 (Cpyrz-3); 11.12 (Cpyrz-5).

2-(Bis((3,5-dimethyl-*1H*-pyrazol-1-yl)methyl)amino) nicotinic acid (**5**)

3-Amino-4-methylnicotinic acid (1 g, 7.24 mmol) and (3,5-dimethyl-*1H*-pyrazol-1-yl)methanol (1.82 g, 14.48 mmol) were mixed together in acetonitrile (20 mL) under reflux for 4 h, and the solvent was evaporated, then recrystallized in diethyl ether, and then filtrated to obtain the final product (0.37 g, 52.1%): mp 78–80 °C (diethyl ether); ^1^H NMR (DMSO-d_6_, 400 MHz) δ ppm: 7.87 (s, 2H, Hpyrn-4 and 6); 6.69 (s, 5H, Hpyrn-5, 2 CH_2_); 5.37 (s, 2H, Hpyrz-4); 2.12 (s, 12H, Hpyrz-3, 5); ^13^C NMR (DMSO-d_6_, 100 MHz) δ ppm: 164.32 (COOH); 163.09 (Cpyrn-1); 154.75 (Cpyrn-4, Cpyrz-3); 110.89 (Cpyrn-5, COOH, Cpyrz-4); 54.24 (2 CH_2_); 20.67 (4 CH_3_). The elemental Analysis was calculated for C_18_H_22_N_6_O_2_ (M.wt 354.43); C- C-61.00, H-6.26, and N-23.71 were the % calculated. C-61.00; H-6.26; and N-23.71 were the % found.

*N,N*-bis((*1H*-pyrazol-1-yl)methyl)-6-methylpyridin-2-amine (**6**) [61]

2-Amino-6-methylpyrimidine (0.5 g, 4.75 mmol) and (*1H*-pyrazol-1-yl)methanol (0.93 g, 9.51 mmol) were mixed together in acetonitrile (20 mL) at room temperature for 4 days, and the solvent was evaporated, then recrystallized in diethyl ether, and then filtrated to obtain the final product (0.19 g, 15.22%): mp 78–80 °C (diethyl ether); FTIR (KBr, cm^−1^): 3300 (N-H); 1614 (C=C); 1532 (C-C); 1473 (C-N); 1281 (C=N); 1082 (N-N); 747 (=C-H); ^1^H NMR (CDCl_3_, 500 MHz) δ ppm: 7.66 (d, 2H, Hpyrz-5, *J_H-H_* = 4–6 Hz); 7.42 (d, 2H, Hpyrz-3, *J_H-H_* = 5–6 Hz); 7.19 (dd, 1H, Hpyrn-2, *J_H-H_* = 6–8 Hz and *J_H-H_* = 8–10 Hz); 6.45 (d, 1H, Hpyrn-3, *J_H-H_* = 4–6 Hz); 6.24 (dd, 2H, Hpyrz-4, *J_H-H_* = 4–6 Hz and *J_H-H_* = 5–6 Hz); 2.33 (s, 3H, Hpyrn-CH_3_); 6.13 (d, 1H, Hpyrn-6, *J_H-H_* = 4–6 Hz); 5.63 (s, 2H, H-CH_2_); ^13^C NMR (CDCl_3_, 125 MHz) δ ppm: 156.49 (Cpyrz-5); 156.09 (Cpyrn-3); 132.46 (Cpyrn-6); 73.79 (Cpyrz-4); 59 (CH_2_); 57.8 (Cpyrn-2); 23.76 (Cpyrn-CH_3_).

*N,N*-bis((3,5-dimethyl-*1H*-pyrazol-1-yl)methyl)-6-methylpyridin-2-amine (**7**) [61]

2-Amino-6-methylpyridine (0.5 g, 4.62 mmol) and (3,5-dimethyl-*1H*-pyrazol-1-yl)methanol (1.17 g, 9.24 mmol) were mixed together in acetonitrile (20 mL) at room temperature for 4 days, and the solvent was evaporated, then recrystallized in diethyl ether, and then filtrated to obtain the final product (1.03 g, 68.7%): mp 96–98 °C (diethyl ether); FTIR (KBr, cm^−1^): 3288 (N-H); 1612 (C=C); 1537 (C-C); 1433 (C-N); 1336 (C=N); 1072 (N-N); 774 (=C-H); ^1^H NMR (CD_2_Cl_2_, 500 MHz) δ ppm: 7.33 (dd, 1H, Hpyrn-3, *J_H-H_* = 4–6 Hz and *J_H-H_* = 5–7 Hz); 6.53 (d, 1H, Hpyrn-2, *J_H-H_* = 4–6 Hz); 6.38 (d, Hpyrn-6, *J_H-H_* = 9–10 Hz); 5.88 (s, 1H, Hpyrz-4); 5.41 (s, 2H, H-CH_2_); 2.48 (s, 3H, Hpyrn-CH_3_); 2.39 and 2.35 (s, 3H, Hpyrz-5); 2.20 and 2.18 (s, 3H, Hpyrz-3); ^13^C NMR (CD_2_Cl_2_, 125 MHz) δ ppm: 156.15 (C-N); 148.33 (Cpyrz-3); 139.56 (Cpyrz-5); 137.56 (Cpyrn-3); 113.25 (Cpyrn-6); 106.96 (Cpyrz-4); 104.69 (1H, Cpyrn-2); 70.67 (2H, CH_2_); 23.90 (3H, Cpyrn-CH_3_); 13.90 (Cpyrz-3); 10.85 (Cpyrz-5).

*N,N*-bis((*1H*-pyrazol-1-yl)methyl)thiazol-2-amine (**8**) [80]

2-Aminothiazole (1 g, 9.98 mmol) and (*1H*-pyrazol-1-yl)methanol (1.96 g, 19.97 mmol) were mixed together in acetonitrile (20 mL) under reflux for 4 h, and the solvent was evaporated, then recrystallized in diethyl ether, and then filtrated to obtain the final product: (2.58 g, 99.41%): mp 84–86 °C (diethyl ether); FTIR (KBr, cm^−1^): 3025 (C-H); 1560 (C=C); 1540 (C-C); 1386 (C-N); 1159 (C=N); 1050 (N-N); 754 (=C-H); 692 (C-S); ^1^H NMR (CD_2_Cl_2_, 500 MHz) δ ppm: 7.79 (d, 2H, Hpyrz-5, *J_H-H_* = 4–6 Hz); 7.55 (s, 1H, Hthi-2); 7.17 (d, 2H, Hpyrz-3, *J_H-H_* = 6–7 Hz); 6.62 (1H, Hthi-5); 6.28 (dd, 2H, Hpyrz-4,); 5.56 (s, 4H, H-CH_2_); ^13^C NMR (CD_2_Cl_2_, 125 MHz) δ ppm: 167.75 (C-N); 139.80 (2H, Cpyrz-3); 138.79 (1H, Cthi-2); 129.53 (Cpyrz-5); 109.76 (1H, Cthi-5); 105.42 (Cpyrz-4); 59.81 (CH_2_).

1-(Bis((*1H*-pyrazol-1-yl)methyl)amino)propan-2-ol (**9**) [81,82,83]

3-Amino propan-2-ol (1 g, 13.31 mmol) and (3,5-dimethyl-*1H*-pyrazol-1-yl)methanol (3.36 g, 26.62 mmol) were mixed together in acetonitrile (20 mL) under reflux for 4 h, and the solvent was evaporated, then recrystallized in diethyl ether, and then filtrated to obtain the final product: yellow oil; ^1^H NMR (DMSO-d_6_, 400 MHz) δ ppm: 7.58 (d, 3H, Hprop-3 and OH, *J_H-H_* = 6–7 Hz); 7.42 (p, 2H, Hpyrz-5, *J_H-H_* = 6–8 Hz); 6.24 (d, 2H, Hpyrz-4, *J_H-H_* = 5–6 Hz); 4.89 (m, 4H, Hprop-2, *J_H-H_* = 5–6 Hz); 4.38 (s, 1H, Hprop-1); 2.46 (d, 2H, CH_2_, *J_H-H_* = 4–6 Hz); 0.97 (s, 3H, H-CH_3_); ^13^C NMR (DMSO-d_6_, 100 MHz) δ ppm: 138.83 (Cpyrz-5); 129.64 (Cpyrz-3); 105.45 (pyrz-4); 66.08 (CH_2_); 65.99 (Cprop-2); 54.05 (CH_2_); 21.55 (CH_3_).

4-(Bis((*1H*-pyrazol-1-yl)methyl)amino)benzonitrile (**10**) [76,84]

4-Aminobenzonitrile (1 g, 8.47 mmol) and (*1H*-pyrazol-1-yl)methanol (1.66 g, 16.94 mmol) were mixed together in acetonitrile (20 mL) under reflux for 4 h, and the solvent was evaporated, then recrystallized in DCM:water (3:1 washed three times in 15:5 mL), and then filtrated to obtain the final product (0.89 g, 96.64%): mp 130–132 °C (diethyl ether); FTIR: 1365.65 cm^−1^ (CN(benzene)); 2218.42 cm^−1^ (C≡N); 2854.74 cm^−1^ (C-H(benzene)); 1455.86 cm^−1^ (C=C(benzene)); 1277.43 cm^−1^ (N-N); 1609.56 cm^−1^ (C=N); 2922.37 cm^−1^ (C-H (CH2) asym); 2854.03 cm^−1^ (C-H (CH2) sym); 822.486 cm^−1^ (H (benzene)); UV-Visible (λ nm): 201.77 (C=N: Transition n→σ*); 206.78 (C=C: Transition π→π*); 245.24 (C=C: Transition π→π*); 250.74 (C=C: Transition π→π*); 264.74 (C=N: Transition n→π *); ^1^H NMR (300 MHz, DMSO-d_6_) δ ppm: 5.994 (s, 2H, H-CH_2_); 6.234 (t, 4H, Hpyrz-4, *J_H-H_* = 2–3 Hz); 6.9015 (d, 2H, Hbz-2, *J_H-H_* = 6.5–7.5 Hz); 7.36 (d, 2H, Hpyrz-3, *J_H-H_* = 9–10 Hz); 7.457 (d, 2H, Hbz-3, *J_H-H_* = 9.5–10.5 Hz); 7.8 (d, 2H, Hpyrz-5, *J_H-H_* = 8–9 Hz); ^13^C NMR (75 MHz, DMSO-d_6_) δ ppm: 65.58 (C-CH_2_); 98.62 (Cbz-1); 105.99 (Cpyrz-4); 113.50 (Cbz-2); 119.90 (Cbz-4); 129.73 (Cpyrz-5); 133.75 (Cbz-3); 139.31 (Cpyrz-3); 150.91 (Cpyrz-5); MS [M^+^] (m/z): 278.86 [M^+^].

4-(Bis((3,5-dimethyl-*1H*-pyrazol-1-yl)methyl)amino)benzonitrile (**11**) [61,76,84]

4-Aminobenzonitrile (1 g, 8.46 mmol) and 2 equiv. of (3,5-dimethyl-*1H*-pyrazol-1-yl)methanol (2.13 g, 16.92 mmol) were mixed together in acetonitrile (20 mL) under reflux for 4 h, and the solvent was evaporated, then recrystallized in DCM:water (3:1 washed three times in 15:5 mL), and then filtrated to obtain the final product (1.22 g, 86.93%): mp 143–145 °C; FTIR (KBr, ν (cm^−1^)): 1365,65 cm^−1^ (CN (benzene)); 2218.21 cm^−1^ (C≡N); 2854.74 cm^−1^ (C-H(benzene)); 1458.23 cm^−1^ (C=C(benzene)); 1271 cm^−1^ (N-N); 1610.61 cm^−1^ (C=N); 2924.15 cm^−1^ (C-H (CH_2_) asym); 2854.74 cm^−1^ (C-H (CH_2_) sym); 2934.24 cm^−1^ (C-H (CH_3_)); 825.56 cm^−1^ (H (benzene)); UV-Visible (λ nm): 207.77 (C=N: Transition n → σ*); 247.27 (C=C: Transition π →π*); 266.73 (C=N: Transition n →π *); ^1^H NMR (300 MHz, DMSO-d_6_) δ ppm: 6.9425 (d, 2H, Hbz-1, *J_H-H_* = 8–9 Hz); 7.485 (d, 2H, Hbz-2, *J_H-H_* = 9–10 Hz); 2.26 (s, 2H, Hpyrz-3); 2.477 (s, 2H, Hpyrz-5); 5.779 (s, 2H, Hpyrz-4); 5.33 (d, 4H, H-CH_2_, *J_H-H_* = 6–7 Hz); ^13^C NMR (75 MHz, DMSO-d_6_) δ ppm: 11.17 (CH_3_-5); 13.78 (CH_3_-3); 62.87 (C-CH_2_); 100.95 (Cbz-1); 106.15 (Cpyrz-4); 113.38 (Cbz-2); 120.02 (Cbz-4); 133.63 (Cbz-3); 139.18 (Cpyrz-5); 146.20 (Cpyrz-3); 151.20 (Cbz-1); MS [M^+^] (m/z)**:** 334.8 [M^+^].

4-(Bis((3,5-dimethyl-*1H*-pyrazol-1-yl)methyl)amino)benzaldehyde (**12**) [61]

4-Aminobenzaldehyde (1 g, 8.25 mmol) and (3,5-dimethyl-*1H*-pyrazol-1-yl)methanol (2.08 g, 16.5 mmol) were mixed together in acetonitrile (20 mL) under reflux for 4 h, and the solvent was evaporated, then recrystallized in diethyl ether, and then filtrated to obtain the final product (1.8 g, 85%): mp 96–98 °C (diethyl ether); ^1^H NMR (DMSO-d_6_, 500 MHz) δ ppm: 9.69 (s, 1H, OH); 7.71 (d, 2H, Hbz-2, *J_H-H_* = 5–6 Hz); 6.81 (d, 2H, Hbz-3, *J_H-H_* = 5–6 Hz); 5.83 (s, 2H, Hpyrz-4); 5.24 (s, 4H, H-CH_2_); 2.25 (s, 6H, CH_3_-5); 2.10 (s, 6H, CH_3_-3); ^13^C NMR (DMSO-d_6_, 125 MHz) δ ppm: 189.85 (C=O); 154.21 (Cbz-1); 145.91 (Cbz-3); 138.68 (Cpyrz-3); 131.52 (Cpyrz-5); 124.51 (Cbz-4); 111.05 (Cbz-2); 13.21 (CH_3_-3); 10.22 (CH_3_-5).

*N,N*-bis((*1H*-pyrazol-1-yl)methyl)-4-nitroaniline (**13**) [61]

4-Nitroaniline (1 g, 7.24mmol) and (*1H*-pyrazol-1-yl)methanol (1.42 g, 14.48 mmol) were mixed together in acetonitrile (20 mL) under reflux for 4 h, and the solvent was evaporated, then recrystallized in diethyl ether, and then filtrated to obtain the final product (0.88 g, 82.05%): mp 86–88 °C (diethyl ether); ^1^H NMR (DMSO-d_6_, 400 MHz) δ ppm: 8.04 (d, 2H, Hbz-2); 7.88 (d, 2H, Hpyrz-5); 7.80 (Hpyrz-3); 6.96 (d, 2H, Hbz-3); 6.28 (t, 2H, Hpyrz-4); 5.61 (s, 4H, H-CH_2_); ^13^C NMR (DMSO-d_6_, 100 MHz) δ ppm: 152.82 (Cbz-1); 139.02 (Cpyrz-3); 138.92 (Cbz-4); 129.44 (Cbz-2); 125.85 (Cpyrz-5); 112.13 (Cbz-3); 105.60 (Cpyrz-4); 57.86 (CH_2_).

4-(Bis((1*H*-pyrazol-1-yl)methyl)amino)phenol (**14**) [66,69,85,86,87]

4-Aminophenol (0.9g, 8.24mmol) and (*1H*-pyrazol-1-yl)methanol (1.62 g, 16.49 mmol) were mixed together in acetonitrile (20 mL) under reflux for 4h, and the solvent was evaporated, then recrystallized in diethyl ether, and then filtrated to obtain the final product (1.8 g, 81.1%): mp 102–104 °C (diethyl ether), FTIR (KBr, cm^−1^): 3113 (O-H); 2300 (C-H); 1659 (C=C); 1509 (C-C); 1393 (C-N); 1183 (C=N); 1039 (N-N); 750 (=C-H), ^1^H NMR (DMSO, 500 MHz) δ ppm: 8.75 (s, 1H, OH); 7.66 (d, 2H, Hbz-5, *J_H-H_* = 4–6 Hz); 7.30 (d, 2H, Hbz-3, *J_H-H_* = 5–6 Hz); 7.15 (d, 2H, Hpyrz-5, *J_H-H_* = 4–6 Hz); 6.96 (d, 2H, Hpyrz-3, *J_H-H_* = 5–7 Hz); 6.31 (dd, 2H, Hpyrz-4, *J_H-H_* = 4–6 Hz and *J_H-H_* = 6–7 Hz); 5.99 (s, 4H, H-CH_2_), ^13^C NMR (DMSO, 125 MHz) δ ppm: 167.79 (Cbz-1); 139.68 (Cbz-3); 138.99 (Cbz-1); 130.48 (Cbz-5); 129.32 (Cpyrz-5); 110.42 (Cpyrz-3); 105.91 (Cpyrz-4); 59.09 (CH_2_).

4-(Bis((1*H*-pyrazol-1-yl)methyl)amino)benzoic acid (**15**)

4-Aminobenzoic acid (1 g, 7.29 mmol) and (*1H*-pyrazol-1-yl)methanol (1.43 g, 14.58 mmol) were mixed together in acetonitrile (20 mL) under reflux for 4 h, and the solvent was evaporated. then recrystallized in diethyl ether, and then filtrated to obtain the final product (1.24 g, 82.05%): mp 164–166 °C (diethyl ether), FTIR (KBr, cm^−1^): 3268 (O-H); 1699 (C=O); 1609 (C=C); 1522 (C-C); 1376 (C-N); 1182 (C=N); 951 (N-N); 764 (=C-H), ^1^H NMR (CD_2_Cl_2_, 500 MHz) δ ppm: 8.02 (d, 2H, Hbz-3, *J_H-H_* = 9–10 Hz); 7.60 (d, 2H, Hpyrz-5, *J_H-H_* = 8–10 Hz); 7.55 (d, 2H, Hpyrz-3, *J_H-H_* = 5–6 Hz); 7.28 (d, 2H, Hbz-2, *J_H-H_* = 5–7 Hz); 6.33 (dd, 2H, Hpyrz-4, *J_H-H_* = 6–8 Hz and *J_H-H_* = 14–15 Hz); 5.88 (s, 4H, H-CH_2_), ^13^C NMR (CD_2_Cl_2_, 125 MHz) δ ppm: 140.03 (Cbz-1); 139.96 (Cpyrz-3); 132 (Cbz-2); 128.88 (Cpyrz-5); 113,74 (Cbz-4); 112.62 (Cbz-3); 106.36 (Cpyrz-4); 66.69 (C-CH_2_). The elemental Analysis was calculated for C_15_H_15_N_5_O_2_ (M.wt 297.12); C-60.60, H-5.09, and N-23.56 were the % calculated. C-60.58; H-5.12; and N-23.45 were the % found.

### 3.3. Biological Evaluation

#### 3.3.1. Antibacterial Assay

The microdilution method with phenol red [88] evaluated the antibacterial effect against four bacterial strains: *Listeria monocytogenes*, *Escherichia coli*, *Staphylococcus aureus, and Citrobacter freundii*. First, the bacterial isolate was cultivated in liquid Luria–Bertani medium (LB) overnight at 37 °C under aeration. Then, a suspension containing 10^6^ CFU/mL of bacteria cells was prepared. Next, an aliquot from this bacterial suspension was added to test tubes containing phenol red medium and the compound to be tested. After an incubation of 24 h at 37 °C, the color of the culture remains red in the absence of growth, indicating that the tested compound has antibacterial activity against the tested strain. However, if there is bacterial growth, the culture becomes yellow due to the acidification of the medium and indicating that the tested compound lacks antibacterial activity against the tested strain. All the experiments were repeated twice for each drug, including the antibiotic streptomycin as a positive control, and means were calculated.

#### 3.3.2. Determination of the Minimum Inhibitory Concentration (MIC) and the Minimum Bactericidal Concentration (MBC)

The MIC (the lowest drug concentration that inhibits bacterial growth after incubation at 37 °C for 24 h) and the MBC (the lowest drug concentration that kills 99% of bacteria after 24 h of incubation) were obtained as described in the literature [89,90].

#### 3.3.3. Antifungal Assay

The prepared ligands were evaluated for their antifungal activity using liquid cell culture against *Saccharomyces cerevisiae* and Candida species: *Candida glabrata* and *Candida albicans*. The growth rate of fungal cells in liquid culture was monitored by absorbance measurements at 600 nm (OD_600_) using a V-1200 spectrophotometer (Shanghai Mapada Instruments Co., Ltd., Shanghai, China). The antifungal activity was described (Bouchal et al., 2019 [88]). Briefly, cells were cultured in the presence and absence of 500 µM of each compound for 24 h, and OD600 measurements were then used to monitor the growth rate. Growth in the presence of a compound was expressed as a percentage relative to the untreated control. All experiments were repeated at least twice, and means were calculated.

### 3.4. Computational Studies

#### 3.4.1. In Silico ADME-Tox Predictions

In silico screening was performed to predict the studied compounds’ properties (absorption, distribution, metabolism, excretion) of the ADME [9,88,91,92,93,94,95,96,97,98,99,100,101,102,103,104]

Additionally, using the SwissADME web tool (www.swissadme.ch/, accessed on 20 April 2022) [105,106,107], lipophilicity (*logP*) was calculated using the Marvin sketch program, while the toxicity predictions were made using the PROTOX online tool (http://tox.charite.de/protox_II/, accessed on 20 April 2022) [108,109,110,111,112,113] based on the functional group similarity of the existing molecules tested in vitro and in vivo in the database. The three most similar compounds were taken for toxicity prediction.

#### 3.4.2. DFT, Molecular Ligand–Protein Docking, and Virtual Screening Studies

The chemical structures of the studied molecules were sketched using Gaussview 6.0, then optimized using the DFT/B3LYP method with 6-31G(d,p) basis sets in the Gaussian 09W software [114]. The docking study performed with New Delhi metallo-β-lactamase was conducted in two different active sites of the crystal structure of NDM-1 at pH 5.5 (Bis-Tris) in a complex with hydrolyzed Ampicillin (**PDB: 5ZGE**) with a resolution of 1.00 Å.

Blind docking/virtual screening was considered to target the previous protein (5ZGE), which was prepared in Autodock 4 [115] default parameters, and the whole protein was used for the grid box (Table S1), with Perl as a launcher for all the ligands in Autodock Vina [116,117,118,119,120].

## 4. Conclusions

Fifteen compounds based on pyrazole derivatives were prepared with good yield and characterized using different physicochemical analyses, such as FTIR, UV-Visible, ^1^H and ^13^C NMR, and MS. These compounds were evaluated for their antifungal and antibacterial activities against several fungal and bacterial strains. None of the compounds had antifungal activity. Interestingly, compounds **12** and **14** displayed intense antibacterial activity. In addition, compound **12** presented excellent antibacterial activity against *E. coli*, *S. aureus,* and *C. freundii*, with inactivity against *L. monocytogenes* and cephalosporins antibiotics. In contrast, compound **14** showed tremendous antibacterial potential against *L. monocytogenes*, with no effect against the other bacterial strains.

Compound **14′**s listericidal activity could be due to the presence of the hydroxyl as a substituent on the phenyl ring, an electron donor group (+M) that causes oxidative stress to the bacterial strain the production of hydroxyl radicals. In contrast, compound **12** has carbonyl as an electron-withdrawing group (-M), methyl as an electron donor group (+I), and a bulky substituent. Furthermore, from the MEP surface analysis, compound **12** only has a higher negative charge value over the carbonyl. In contrast, compound **14,** similar to the antibiotics cefotaxime and Ampicillin, has a close negative-positive (δ^+^-δ^−^) higher charge value over the hydroxyl and the acidic function.

From ADME and toxicity predictions, compounds **12** and **14** have no violations of Lipinski’s rule of five, better than streptomycin, with three violations, and cefotaxime, with one offense. In contrast, compounds **12** and **14** have lower predicted LD_50_ than Ampicillin and cefotaxime, with less toxicity (class 4) than streptomycin (class 3), even though they are both active as carcinogens with mutagenic activity for compound **14** and have no binding probability with all the toxicity targets better than Ampicillin and cefotaxime, which have probable binding with toxicity targets.

From the docking results, compound **12** has a better affinity with both active protein sites, which have an H-acceptor bond, than compound **14**, with ligand exposure. However, Ampicillin and cefotaxime still have the best values, with more H-donors and H-acceptors; otherwise, compound **12** is hydrolyzed by NDM1 hydrolysis and inactivates its antibacterial activity against *Listeria monocytogenes* due to the presence of the carbonyl function, which is common in all β-lactam antibiotics. On the other hand, compound **14** has listericidal activity with a specific mechanism that needs more computational studies and biological assays for prediction; from blind docking/virtual screening studies, the mode of binding interaction is the same as that of LYS216, which is bound with the nitrogen of pyrazole for the ligand **12** and the oxygen for Ampicillin with the same binding affinity of −7.1 Kcal/mol. On the other hand, ligand **14** has a lower −7.0 Kcal/mol value with amino acids such as ILE203 and LYS242.

## Figures and Tables

**Table 1:** The antibiotic activity of the active synthesized pyrazole ligands was determined using the broth macro dilution assay and the phenol red indicator.

Compound	*L. monocytogenes*	*S. aureus*	*E. coli*	*C. freundii*
**12**	− − −	+ + +	+ + +	+ + +
**14**	+ + +	− − −	− − −	− − −
**Streptomycin**	+ + +	+ + +	+ + +	+ + +

The compounds and the positive control (streptomycin) were used at 500 µM and 50 mg/L concentrations. All experiments were repeated three times, and the result obtained for each time is presented. (−): no inhibition of bacterial growth; (+): inhibition of bacterial growth.

**Table 2:** MIC and MBC values in mg/L of the studied compounds **12** and **14** against the used bacterial strains.

		12	14
***L. monocytogenes***	**MIC**	-	134.6 ± 0
	**MBC**	-	242.3 ± 0
	**MBC/MIC**	-	1.2
***S. aureus***	**MIC**	168.7 ± 0	-
	**MBC**	202.4 ± 0	-
	**MBC/MIC**	1.2	-
***E. coli***	**MIC**	134.9 ± 0	-
	**MBC**	134.9 ± 0	-
	**MBC/MIC**	1	-
***C. freundii***	**MIC**	168.7 ± 0	-
	**MBC**	236.2 ± 0	-
	**MBC/MIC**	1.4	-

**MIC:** Minimum inhibition concentration; **MBC:** Minimum bactericidal concentration.

**Table 3:** The physicochemical properties of the compounds **12**, **14,** and the drugs streptomycin, Ampicillin, and cefotaxime.

Compound	MW	logP	HDO	HAC	NRO	TPSA (Å^2^)
**12**	337.42	3.09	0	3	6	55.95
**14**	269.30	1.22	1	3	5	59.11
**streptomycin**	581.57	−6.65	14	15	11	331.43
**ampicillin**	349.40	0.26	3	5	5	138.03
**cefotaxime**	455.47	−0.73	3	9	9	227.05

**Table 4:** The binding affinity values of the compounds **12**, **14**, and ampicillin within the two NDM1 chains A and B.

Compound	NDM1 (A)	NDM1 (B)
	Binding Affinity in kcal/mol	Binding Affinity in kcal/mol
**12**	−6.0075	−6.6776
**14**	−5.5411	−5.6752
**ampicillin**	−6.9737	−6.7344

**Table 5:** Docking results of the compounds **12**, **14,** and ampicillin in the two NDM1 chains A and B.

	NDM1 (A)		NDM1 (B)	
Compound	Interaction L-AA	Bond Length (Å)	Interaction L-AA	Bond Length (Å)
**12**	O47---ND1 His 122: **H-acceptor**	3.08	O47---NZ Lys211: **H-acceptor**	2.98
	O47---NE2 His 189: **H-acceptor**	2.9	5-ring---r-ring His 122: **pi-pi**	3.62
**14**	6-ring---N Asn 220: **pi-H**	3.9	6-ring---N Asn220: **pi-H**	3.89
	5-ring---5-ring His 122: **pi-pi**	3.6	5-ring---5-ring His 122: **pi-pi**	3.64
**ampicillin**	OXT45---OD1 Asp124: **H-donor**	2.9		
	O1 1---NZ Lys211: **H-acceptor**	3.37		
	O2 3---NZ Lys211: **H-acceptor**	2.98	OXT45---OD1 Asp124: **H-donor**	2.99
	O3 28---N Asp124: **H-acceptor**	3.55	O1 1---NZ Lys211: **H-acceptor**	2.88
	C16 12---5-ring His 250: **H-pi**	4.07	O3 28---N Asp124: **H-acceptor**	3.38
	6-ring---N Gln 123: **pi-H**	4.08		
	6-ring---CB Gln 123: **pi-H**	3.78		

**Table 6:** Binding affinity and L–AA interaction of the ligands **12** and **14** and ampicillin with the chosen target, NDM1.

Compound	Binding Affinity in kcal/mol	Interaction L–AA (Hydrogen Bonds Only)
**12**	−7.1	N(pyrazole)---LYS216
**14**	−7.0	OH----ILE203 N(pyrazole)---LYS242
**ampicillin**	−7.1	NH2---SER251 NH2---HIS250 O---LYS216

## Data Availability

Data are contained within the article and supplementary material.

## Supplementary Materials

The following are available online at https://www.mdpi.com/article/10.3390/ph15070803/s1, Table S1: Blind docking/virtual screening as Perl configuration and GRID box parameters; File S1: ^1^HNMR and ^13^CNMR spectra of compounds.
