# Supplementary material for: New N-Alkylated Heterocyclic Compounds as Prospective NDM1 Inhibitors: Investigation of In Vitro and In Silico Properties
Source: Pharmaceuticals (Basel). 2022 Jun 28;15(7):803. doi: 10.3390/ph15070803 (PMC9322059; doi:10.3390/ph15070803)
Supplement: Supplementary file 1 [file pharmaceuticals-15-00803-s001.zip › File S1.pdf]

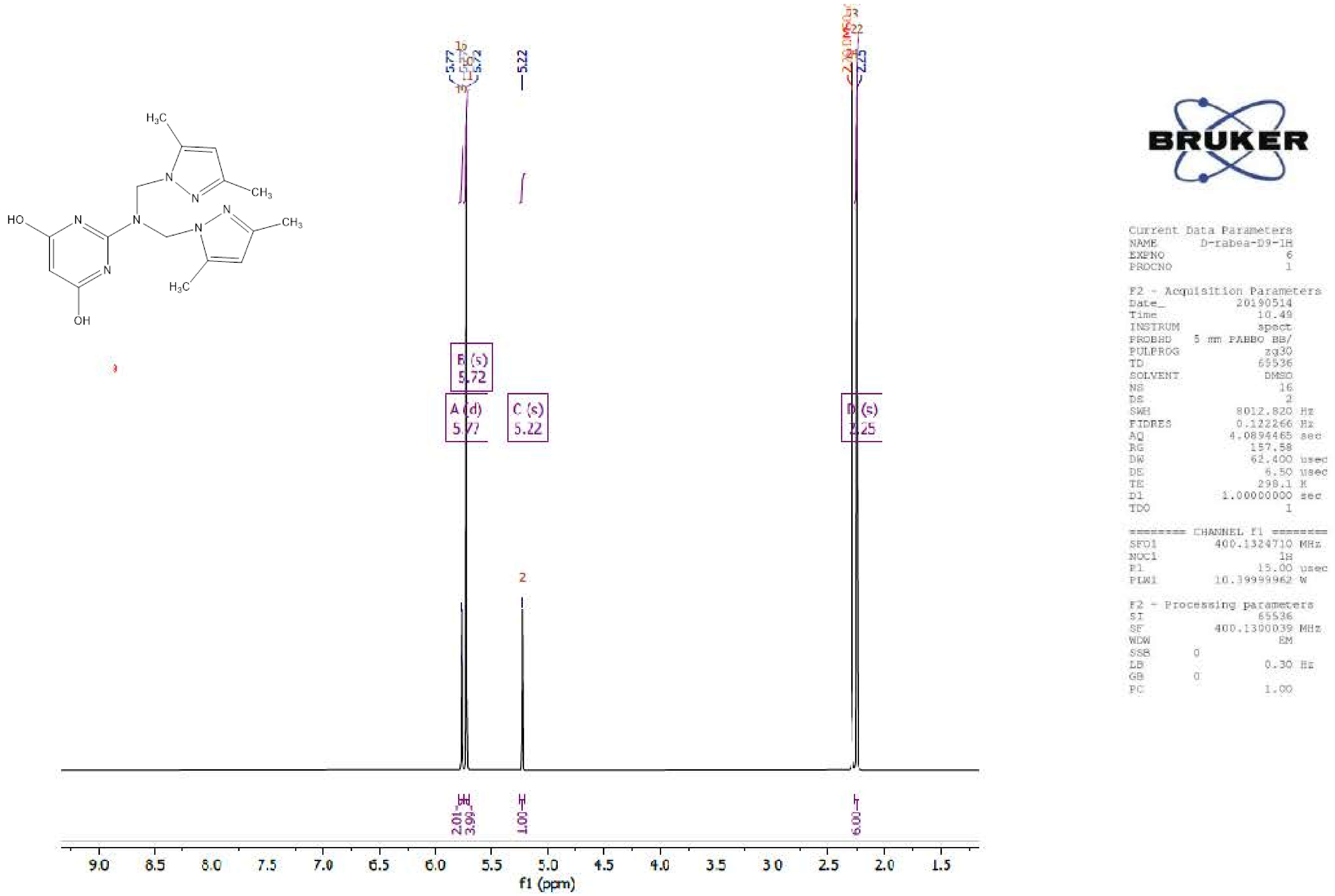

HO
OH
N
H3C
CH3
5.77
5.72
5.22
2.25
B (s)
5.72
A (d)
5.77
C (s)
5.22
D (s)
2.25
2.01
3.99
1.00
6.00
9.0 8.5 8.0 7.5 7.0 6.5 6.0 5.5 5.0 4.5 4.0 3.5 3.0 2.5 2.0 1.5
f1 (ppm)
BRUKER
Current Data Parameters
NAME D-rabea-D9-1H
EXPNO 6
PROCNO 1
F2 - Acquisition Parameters
Date_ 20190514
Time 10.49
INSTRUM spect
PROBHD 5 mm PABBO BB/
PULPROG zg30
TD 65536
SOLVENT DMSO
NS 16
DS 2
SWH 8012.820 Hz
FIDRES 0.122266 Hz
AQ 4.0894465 sec
RG 157.58
DW 62.400 usec
DE 6.50 usec
TE 298.1 K
D1 1.00000000 sec
TD0 1
======== CHANNEL f1 ========
SFO1 400.1324710 MHz
NUC1 1H
P1 15.00 usec
PLW1 10.39999962 W
F2 - Processing parameters
SI 65536
SF 400.1300039 MHz
WDW EM
SSB 0
LB 0.30 Hz
GB 0
PC 1.00

Compound 1

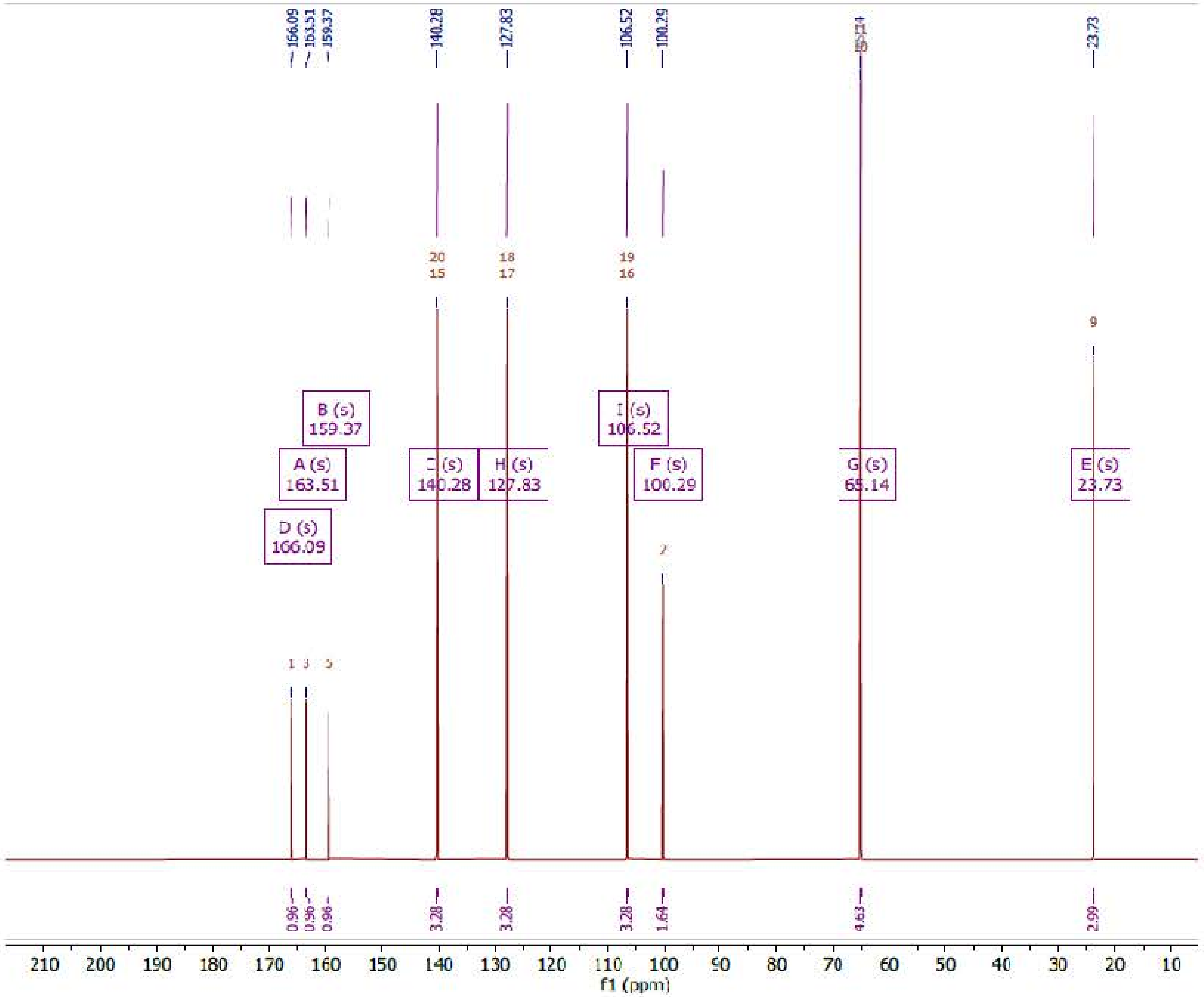


```
Current Data Parameters
NAME          D-rabea-D9-1H
EXPNO                     6
PROCNO                    1

F2 - Acquisition Parameters
Date_              20190514
Time                  10.49
INSTRUM               spect
PROBHD    5 mm PABBO BB/
PULPROG                zg30
TD                    65536
SOLVENT                DMSO
NS                       16
DS                        2
SWH                8012.820 Hz
FIDRES             0.122266 Hz
AQ                4.0894465 sec
RG                   157.58
DW                   62.400 usec
DE                     6.50 usec
TE                    298.1 K
D1               1.00000000 sec
TD0                       1

======== CHANNEL f1 ========
SFO1            400.1324710 MHz
NUC1                     1H
P1                    15.00 usec
PLW1            10.39999962 W

F2 - Processing parameters
SI                    65536
SF              400.1300039 MHz
WDW                      EM
SSB       0
LB                     0.30 Hz
GB        0
PC                     1.00
```

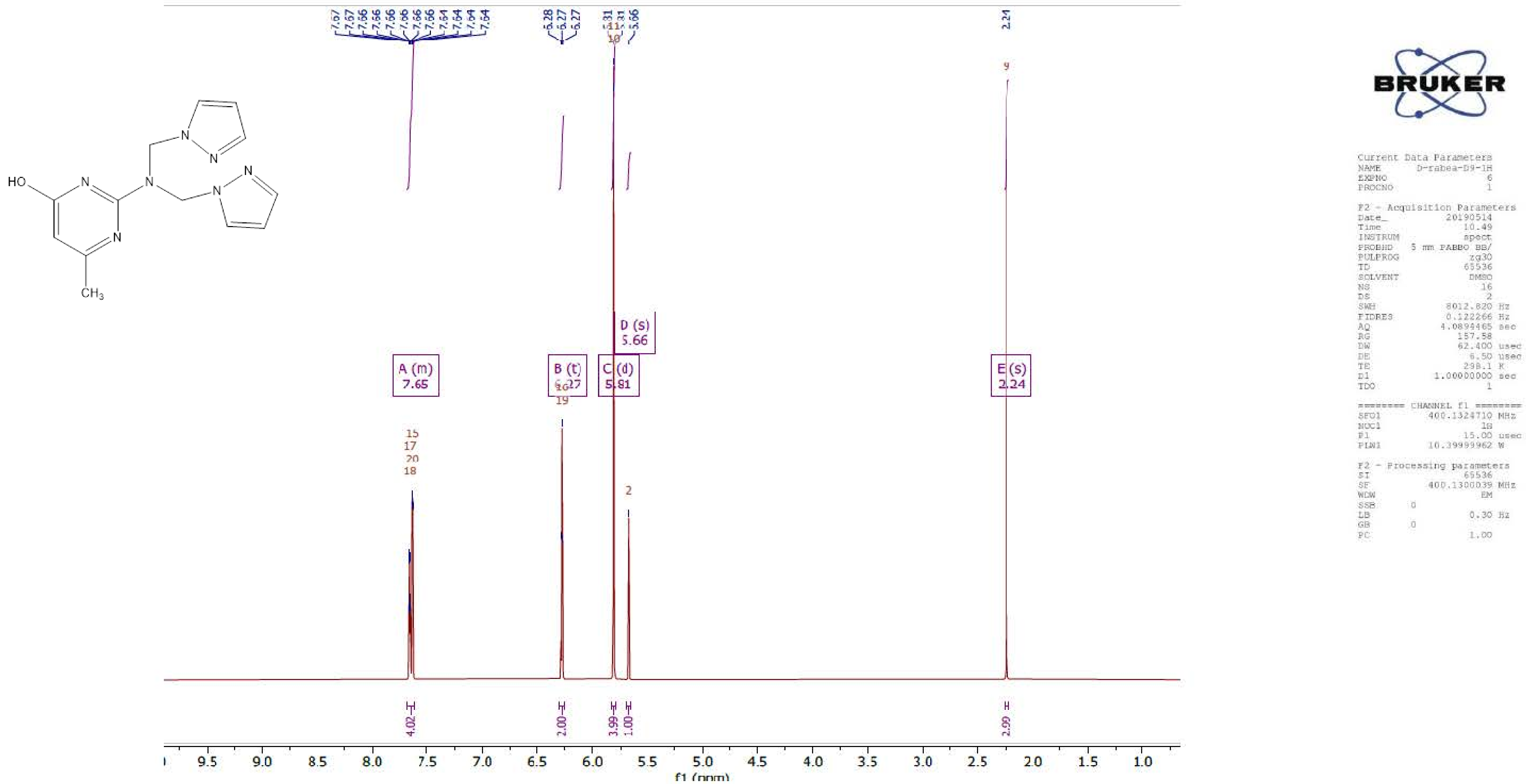

HO
N
N
N
N
N
N
N
CH3
A (m)
7.65
B (t)
6.27
C (d)
5.81
D (s)
5.66
E (s)
2.24
4.02
2.00
3.99
1.00
2.99
f1 (ppm)
BRUKER
Current Data Parameters
NAME D-rabea-D9-1H
EXPNO 6
PROCNO 1
F2 - Acquisition Parameters
Date_ 20190514
Time 10.49
INSTRUM spect
PROBHD 5 mm PABBO BB/
PULPROG zg30
TD 65536
SOLVENT DMSO
NS 16
DS 2
SWH 8012.820 Hz
FIDRES 0.122266 Hz
AQ 4.0894465 sec
RG 157.58
DW 62.400 usec
DE 6.50 usec
TE 298.1 K
D1 1.00000000 sec
TD0 1
======== CHANNEL f1 ========
SFO1 400.1324710 MHz
NUC1 1H
P1 15.00 usec
PLW1 10.39999962 W
F2 - Processing parameters
SI 65536
SF 400.1300039 MHz
WDW EM
SSB 0
LB 0.30 Hz
GB 0
PC 1.00

Compound 2

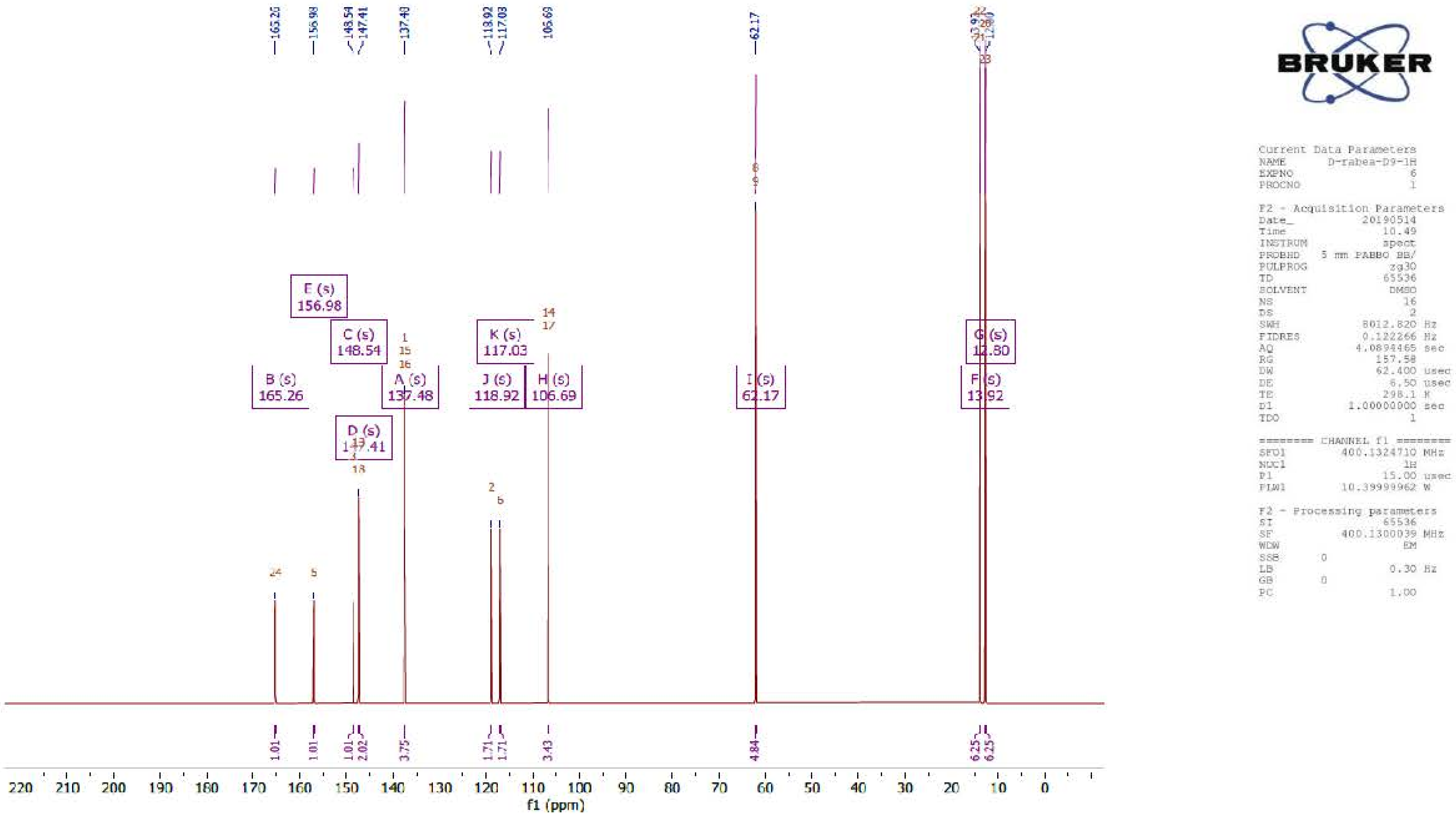
BRUKER
Current Data Parameters
NAME D-rabea-D9-1H
EXPNO 6
PROCNO 1
F2 - Acquisition Parameters
Date_ 20190514
Time 10.49
INSTRUM spect
PROBHD 5 mm PABBO BB/
PULPROG zg30
TD 65536
SOLVENT DMSO
NS 16
DS 2
SWH 8012.820 Hz
FIDRES 0.122266 Hz
AQ 4.0894465 sec
RG 157.58
DW 62.400 usec
DE 6.50 usec
TE 298.1 K
D1 1.00000000 sec
TD0 1
======== CHANNEL f1 ========
SFO1 400.1324710 MHz
NUC1 1H
P1 15.00 usec
PLW1 10.39999962 W
F2 - Processing parameters
SI 65536
SF 400.1300039 MHz
WDW EM
SSB 0
LB 0.30 Hz
GB 0
PC 1.00
B (s) 165.26
E (s) 156.98
C (s) 148.54
D (s) 147.41
A (s) 137.48
J (s) 118.92
K (s) 117.03
H (s) 106.69
I (s) 62.17
F (s) 13.92
G (s) 12.80
f1 (ppm)

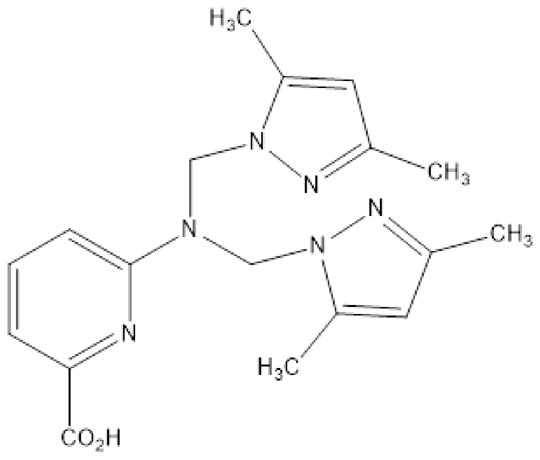

H_3C
N
N
CH_3
N
N
N
CH_3
N
H_3C
CO_2H

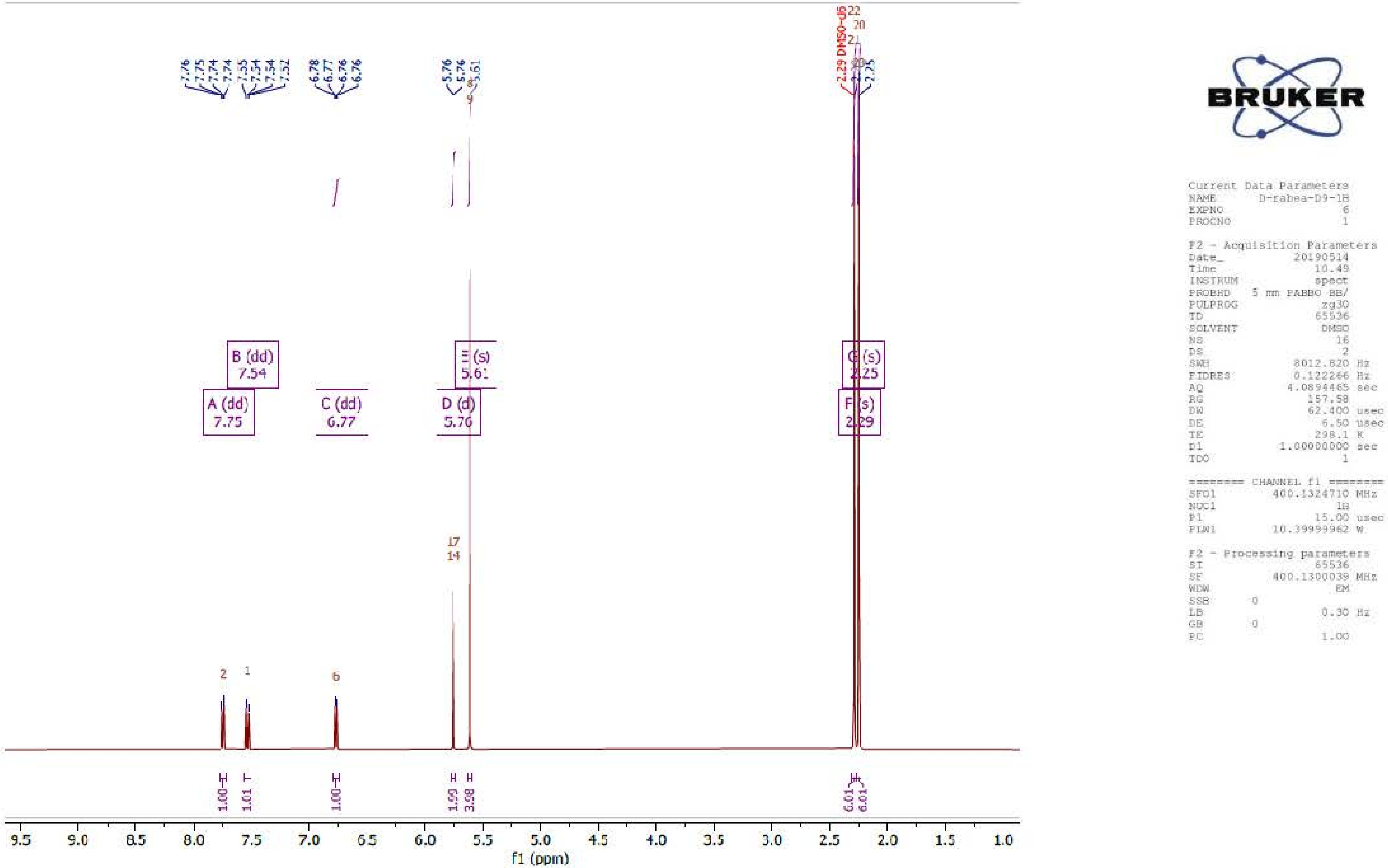

A (dd) 7.75
B (dd) 7.54
C (dd) 6.77
D (d) 5.76
E (s) 5.61
F (s) 2.29
G (s) 2.25
2.29 DMSO-d6
1.00
1.01
1.00
1.99
3.98
6.01
6.01
f1 (ppm)
BRUKER
Current Data Parameters
NAME D-rabea-D9-1H
EXPNO 6
PROCNO 1
F2 - Acquisition Parameters
Date_ 20190514
Time 10.49
INSTRUM spect
PROBHD 5 mm PABBO BB/
PULPROG zg30
TD 65536
SOLVENT DMSO
NS 16
DS 2
SWH 8012.820 Hz
FIDRES 0.122266 Hz
AQ 4.0894465 sec
RG 157.58
DW 62.400 usec
DE 6.50 usec
TE 298.1 K
D1 1.00000000 sec
TD0 1
======== CHANNEL f1 ========
SFO1 400.1324710 MHz
NUC1 1H
P1 15.00 usec
PLW1 10.39999962 W
F2 - Processing parameters
SI 65536
SF 400.1300039 MHz
WDW EM
SSB 0
LB 0.30 Hz
GB 0
PC 1.00

Compound 5

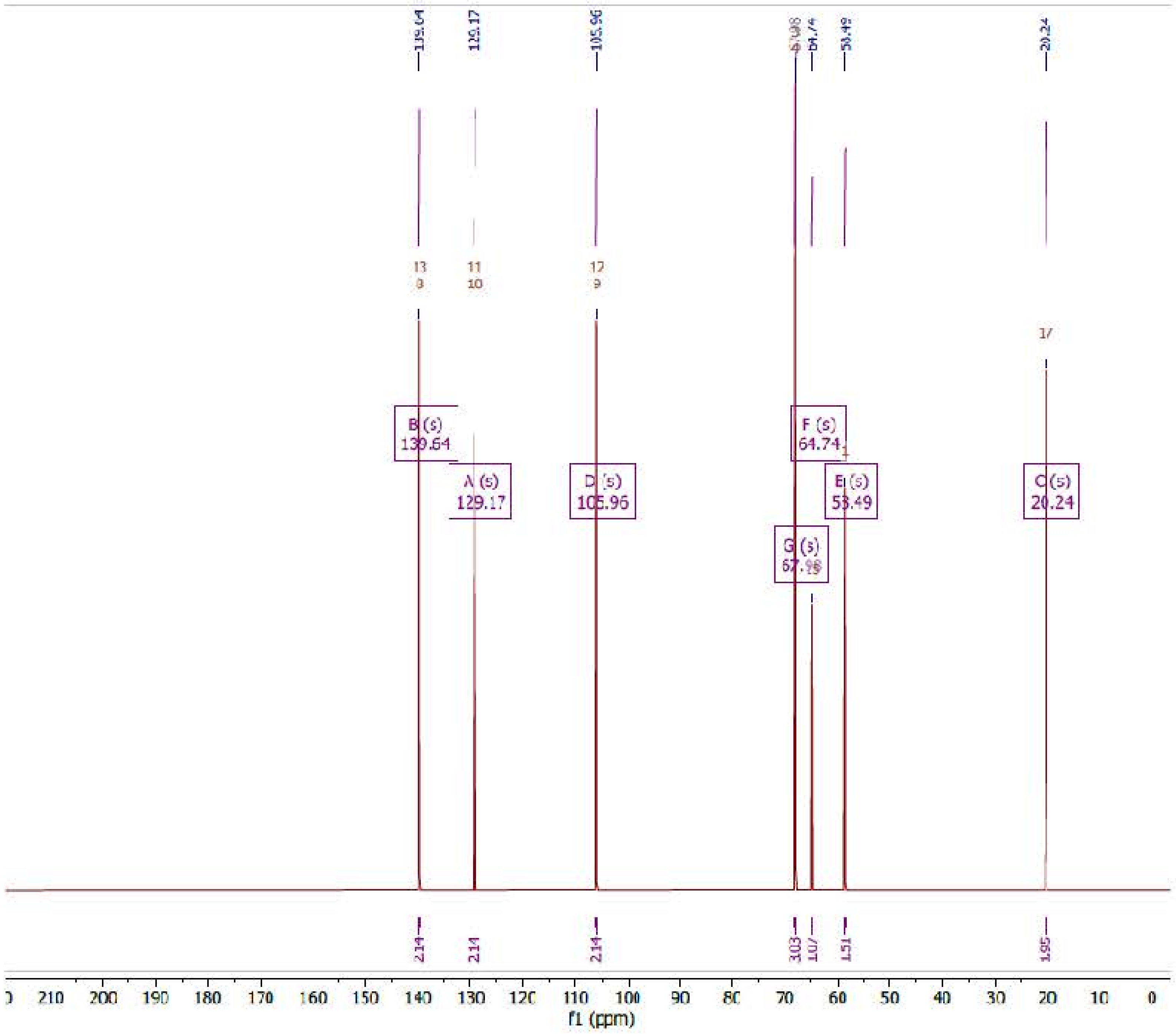


```
Current Data Parameters
NAME        D-rabea-D9-1H
EXPNO                   6
PROCNO                  1

F2 - Acquisition Parameters
Date_            20190514
Time                10.49
INSTRUM             spect
PROBHD   5 mm PABBO BB/
PULPROG              zg30
TD                  65536
SOLVENT              DMSO
NS                     16
DS                      2
SWH              8012.820 Hz
FIDRES           0.122266 Hz
AQ              4.0894465 sec
RG                 157.58
DW                 62.400 usec
DE                   6.50 usec
TE                  298.1 K
D1             1.00000000 sec
TD0                     1

======== CHANNEL f1 ========
SFO1          400.1324710 MHz
NUC1                   1H
P1                  15.00 usec
PLW1          10.39999962 W

F2 - Processing parameters
SI                  65536
SF            400.1300039 MHz
WDW                    EM
SSB        0
LB                   0.30 Hz
GB         0
PC                   1.00
```

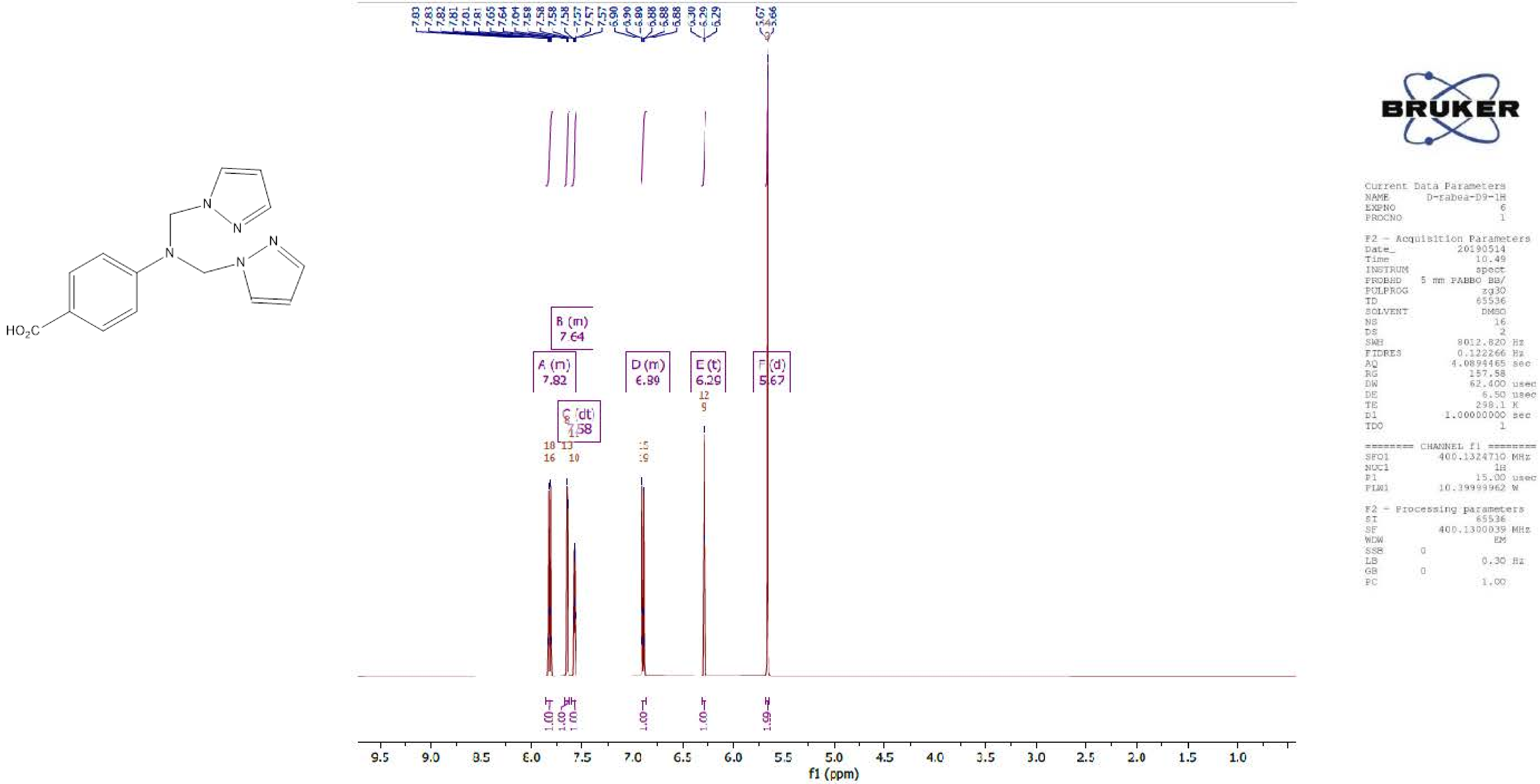
HO2C
N
A (m)
7.82
B (m)
7.64
C (dt)
7.58
D (m)
6.89
E (t)
6.29
F (d)
5.67
1.00
1.00
1.00
1.00
1.00
1.99
9.5 9.0 8.5 8.0 7.5 7.0 6.5 6.0 5.5 5.0 4.5 4.0 3.5 3.0 2.5 2.0 1.5 1.0
f1 (ppm)
Current Data Parameters
NAME D-rabea-D9-1H
EXPNO 6
PROCNO 1
F2 - Acquisition Parameters
Date_ 20190514
Time 10.49
INSTRUM spect
PROBHD 5 mm PABBO BB/
PULPROG zg30
TD 65536
SOLVENT DMSO
NS 16
DS 2
SWH 8012.820 Hz
FIDRES 0.122266 Hz
AQ 4.0894465 sec
RG 157.58
DW 62.400 usec
DE 6.50 usec
TE 298.1 K
D1 1.00000000 sec
TD0 1
======== CHANNEL f1 ========
SFO1 400.1324710 MHz
NUC1 1H
P1 15.00 usec
PLW1 10.39999962 W
F2 - Processing parameters
SI 65536
SF 400.1300039 MHz
WDW EM
SSB 0
LB 0.30 Hz
GB 0
PC 1.00

Compound 15

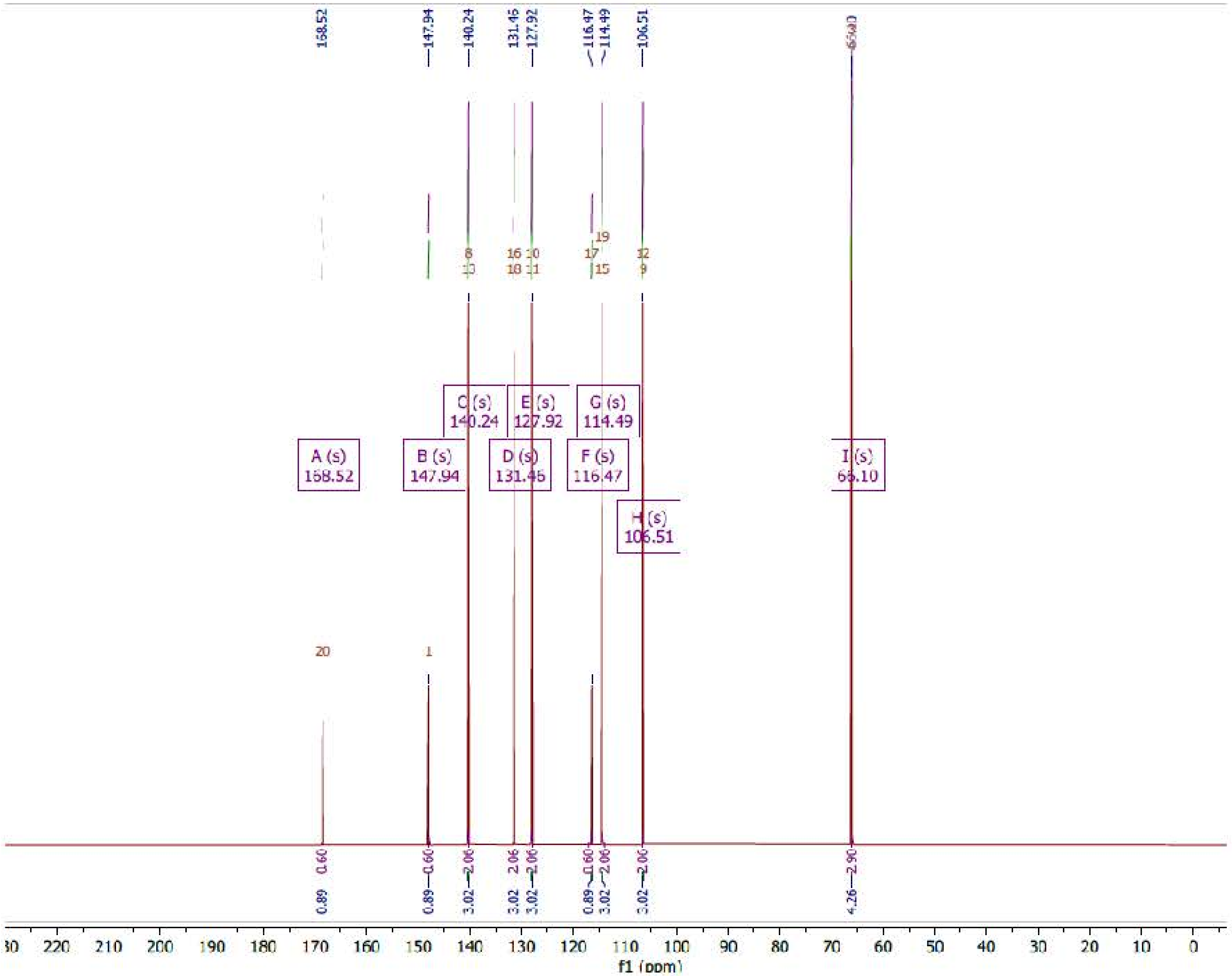

BRUKER

Current Data Parameters

NAME	D-rabea-D9-1H
EXPNO	6
PROCNO	1

F2 - Acquisition Parameters

Date_	20190514
Time	10.49
INSTRUM	spect
PROBHD	5 mm PABBO BB/
PULPROG	zg30
TD	65536
SOLVENT	DMSO
NS	16
DS	2
SWH	8012.820 Hz
FIDRES	0.122266 Hz
AQ	4.0894465 sec
RG	157.58
DW	62.400 usec
DE	6.50 usec
TE	298.1 K
D1	1.00000000 sec
TD0	1

======== CHANNEL f1 ========

SFO1	400.1324710 MHz
NUC1	1H
P1	15.00 usec
PLW1	10.39999962 W

F2 - Processing parameters

SI	65536
SF	400.1300039 MHz
WDW	EM
SSB	0
LB	0.30 Hz
GB	0
PC	1.00
