# Supplementary material for: New N-Alkylated Heterocyclic Compounds as Prospective NDM1 Inhibitors: Investigation of In Vitro and In Silico Properties
Source: Pharmaceuticals (Basel). 2022 Jun 28;15(7):803. doi: 10.3390/ph15070803 (PMC9322059; doi:10.3390/ph15070803)
Supplement: Supplementary file 1 [file pharmaceuticals-15-00803-s001.zip › Table S1.pdf]

Table S1. Blind docking/virtual screening as Perl configuration and GRID box parameters.

<table>
<tr><td>Perl configuration</td><td>#!/usr/bin/perl
print“Ligand_file:\t”;
$ligfile=<STDIN>;
chomp $ligfile;
open (FH,$ligfile)||die “Cannot open file\n”;
@arr_file=<FH>;
for($i=0;$i<@arr_file;$i++)
{
print“@arr_file[$i]\n”;
@name=split(/\./,@arr_file[$i]);
}
for($i=0;$i<@arr_file;$i++)
{
chomp @arr_file[$i];
print“@arr_file[$i]\n”;
system(“vina.exe –config conf_vs.txt –ligand @arr_file[$i]
–log @arr_file[$i]_log.log”);
}</td></tr>
<tr><td>GRID box parameters</td><td>• center_x = 2.863, center_y = 33.906, center_z = 116.625
• size_x = 54, size_y = 86, size_z = 54
• num_modes = 10; energy_range = 4</td></tr>
</table>
